# Decrements of mobility and power in recreationally active septuagenarians is related to loss of force, but not slowing of the muscle: a 5-year longitudinal study

**DOI:** 10.1007/s00421-023-05160-0

**Published:** 2023-02-28

**Authors:** James Cameron, Jamie S. McPhee, David A. Jones, Hans Degens

**Affiliations:** 1 Department of Health Professions, Manchester Metropolitan University, Manchester, UK; 2 School of Healthcare Science, Manchester Metropolitan University, Manchester, UK; 3 Department of Sport and Exercise Sciences, Manchester Metropolitan University, Manchester, UK; 4 Institute of Sport Science and Innovations, Lithuanian Sports University, Kaunas, Lithuania

**Keywords:** Physical function, Muscle power, Ageing

## Abstract

A lesser 6-min walk distance (6MWD) and timed up-and-go (TUG) in old compared with young adults was previously linked to slowing of muscle contractile properties. The purpose of the present study was to determine whether any further reductions in 6MWD and TUG over a 5-year period in septuagenarians are associated with further slowing of muscle contractile properties. We measured muscle function by a countermovement jump, isometric maximal knee extensor strength (MVC) on a dynamometer and quadriceps muscle size by magnetic resonance imaging (MRI) in 17 older women (71.1 ± 2.8 y) and 17 older men (71.3 ± 4.1y). Performance in TUG and 6MWD were reduced over the 5-year period, irrespective of sex (*P* < 0.001), and both were correlated with power at both baseline and follow-up (*R* ≥ 0.53; *P* ≤ 0.001). Jump take-off velocity (V_CMJ_) was slower at follow-up (*P* < 0.01) and correlated with 6MWD and TUG at both baseline and follow-up in both sexes (*R* ≥ 0.54; *P* ≤ 0.001). However, the relationship between ‘body mass: maximal muscle force ratio’ with V_CMJ_ was not significantly changed, indicating that the lower V_CMJ_ was attributable to muscles working at a higher relative load, hence a lower part of the force–velocity relationship, due to a reduction in MVC (body mass had not changed significantly), rather than slowing of the muscle. The lower V_CMJ_ in women than men (*P* < 0.001) was likewise attributable to a lower MVC rather than slower contractile properties in women. In conclusion, the decrement in 6MWD and TUG in septuagenarians is due to a loss of muscle mass, rather than further loss of muscle quality.

## Introduction

Globally, in 2017 there were an estimated 962 million people over the age of 60, which is expected to rise to 2.1 billion people by 2050 (United-Nations 2017). Mobility limitations leading to loss of independence are now affecting a large proportion of older people (35% aged 70 and over) (Freiberger et al. 2020), placing a significant stress on healthcare resources. The age-related reductions in musculoskeletal function (dynapenia) and mass (sarcopenia) contribute to decreased mobility and may ultimately lead to loss of independence and quality of life (Evans and Campbell 1993; Goodpaster et al. 2006; McPhee et al. 2016).

The difficulties of many older people to perform daily life activities, such as transitions from sitting to standing and walking, are most pronounced amongst people with low skeletal muscle mass and muscle weakness (Rantanen et al. 1999; Reid and Fielding 2012). However, the associations of lower limb muscle mass with physical function in older age are weak (Bijlsma et al. 2014; Janssen et al. 2002). This is perhaps somewhat surprising, but partly explicable by the common observation that muscle force declines proportionally more than muscle size with age (Degens et al. 2009; McPhee et al. 2018; Morse et al. 2005). Another factor that is not considered in these associations is that most contractions during daily movement are not isometric, but rather shortening (or lengthening) contractions that require power, the product of force and velocity. This is significant, as a lower force generating capacity combined with slower contractile properties due to preferential atrophy of fast fibres (Barnouin et al. 2017) will exacerbate the ageing-related loss of power (Degens 2019﻿; Larsson et al. 2019). In line with this, previous studies have shown that muscle force and power are more closely correlated to physical function than muscle mass in older people (Bean et al. 2002; Buford et al. 2012; Maden-Wilkinson et al. 2015; Reid and Fielding 2012).

Another consideration is that general physical function requires good balance and postural control. Balance is commonly measured as the ability to stand on one leg with eyes open or closed. Performance in this test is dramatically poorer in older age compared to those in their mid-20s (Onambele et al. 2006) and is associated with slower walking speed and sit-to-stand performance (Maden-Wilkinson et al. 2015; Montgomery et al. 2020). Taken together, this suggests that in addition to low muscle mass and power, poor balance also contributes to lower physical capability in the older population.

Athletic prowess and muscle power generally peak during the mid-20’s (Berthelot et al. 2012; Rittweger et al. 2009), but upon entering the 4th decade there is a noticeable and progressive decline in performance and strength (Janssen et al. 2000; Ocana et al. 2021) with an accelerated decline after the age of 70 (Ganse et al. 2018; Hughes et al. 2001; Nikolaidis 2018). Also in the non-athletic population, accelerated muscle wasting and weakness have been observed after the age of 70 years (Delmonico et al. 2009; Deschenes 2004; Frontera et al. 2000, 2008). In addition, we found evidence that the pattern of muscle ageing may change over time, where the initial ageing-related reduction in MVC is attributable to loss of muscle mass and specific tension, while the further reduction in MVC and peak jumping power in septuagenarians and octogenarians was due to a further loss of muscle mass, but not specific tension (Degens et al. 2021; McPhee et al. 2018).

In 2015 (Maden-Wilkinson et al. 2015) we published the results of a cross-sectional study of young adults (average age 23 years) and older men and women (average age 72 yrs). In that study there was evidence of an association between lower power, determined from a countermovement jump, lower Timed Up-and-Go (TUG) and 6-min walking distance (6MWD) performance, that appeared to be the result of both weaker and intrinsically slower muscles of the old than young-adult people. It remains to be seen to what extent any additional reduction in performance of TUG and 6MWD in recreationally active older people can be ascribed to further slowing and/or weakening of the muscle.

Therefore, the aim of the present study was to determine changes in walking speed (6MWD) and performance in a short mobility task (TUG) over a 5-year follow-up in relation to changes in muscle maximal force, power and balance in septuagenarians. We hypothesised that declining physical function during ageing is more strongly associated with reduced balance and power than with muscle mass, and that any additional decrement in performance is primarily related to a further intrinsic slowing of the muscle.

## Methods

### Participants and ethical approval

**Fig. 1:**
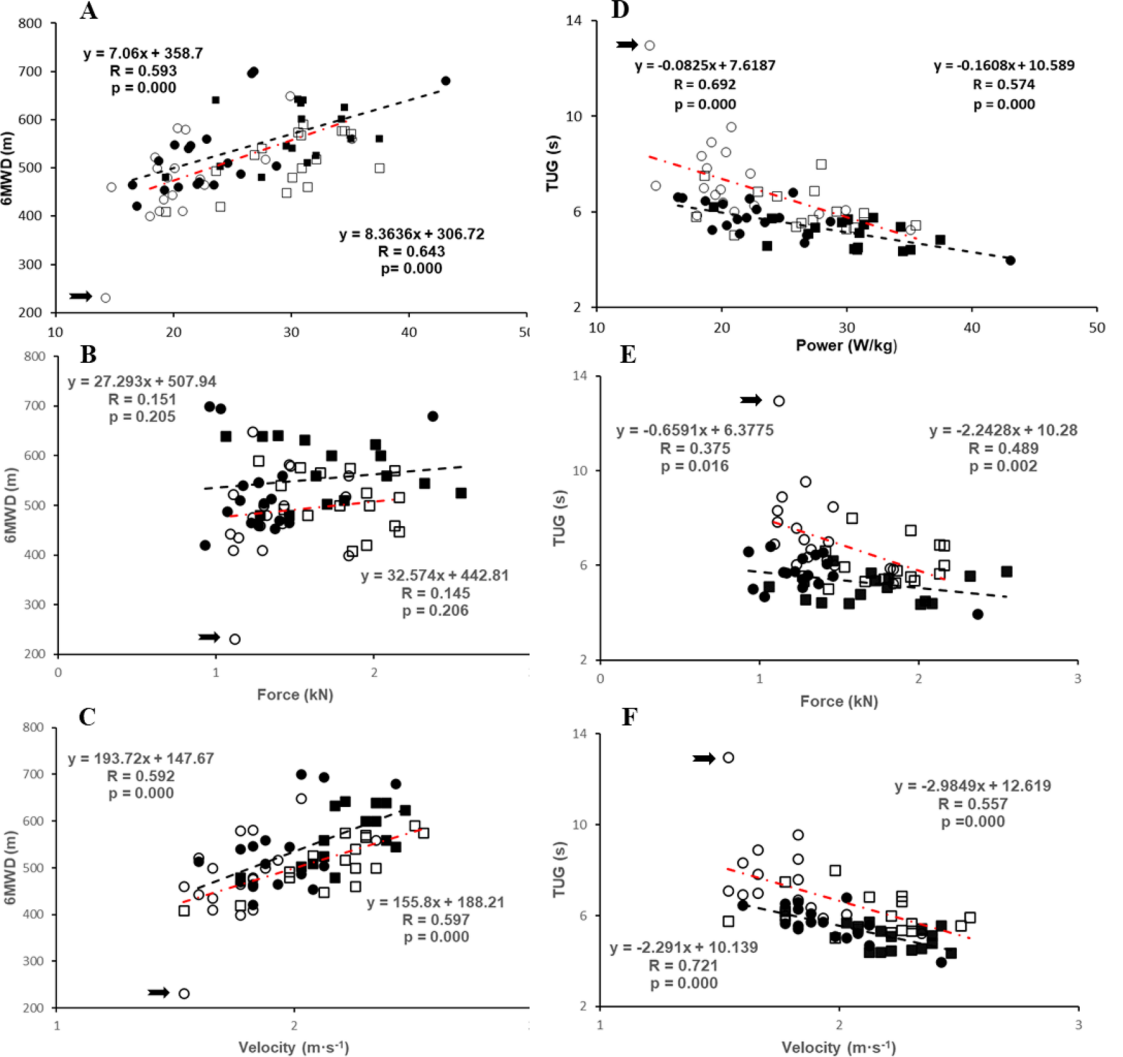
The relationship between (**A–C**) 6-min walking distance (6MWD; m) and (**D–F**) timed up-and-go (TUG; s), with (**A**, **D**) power (W·kg^−1^), (**B**, **E**) maximum voluntary contraction / body mass (MVC·BM^−1^; Nm·kg^−1^), (**C**, **F**) take-off velocity during the countermovement jump (V_CMJ_; m·s^−1^). ■: men and ●: women at baseline, and □: men and ○: women at follow-up. –-: regression line at baseline; red —: regression line at follow-up. Regression equation left at baseline, right at follow-up. Arrow indicates woman with poor performance in follow-up

**Fig. 2:**
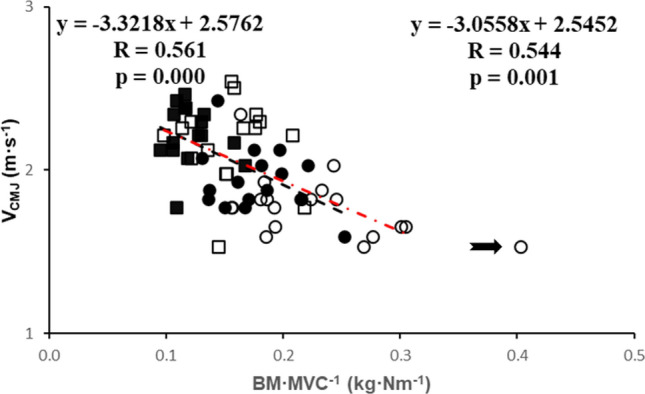
Body mass/MVC (kg·Nm^−1^) versus velocity (m·s^−1^). ■: men and ●: women at baseline, and □: men and ○: women at follow-up. ---: regression line at baseline; red -·-: regression line at follow-up. Regression equation left at baseline, right at follow-up. Arrow indicates woman with poor performance in follow-up

The study received approval from the local ethics committee and was performed in accordance to the declaration of Helsinki. Participants were recruited from a subgroup in the framework of the MYOAGE study (www.myoage.eu) (McPhee et al. 2013). Thirty-five participants returned 5 years following the initial cross-sectional study from 2009 to 2012 (base line). The follow-up study was conducted between May 2015 and October 2015. The data of one woman are presented in figures, but not included in statistical analyses as her performance in the 6-min walk and timed up-and-go tests at follow-up was more than 3 standard deviations below the average performance of the women at follow-up (see her performance indicated with arrows in Figs. 1 and 2).

The characteristics of the included participants are presented in Table 1. Written informed consent was obtained at both baseline and follow-up from each participant. Exclusion criteria were: institutionalisation, unable to complete 250 m walking unassisted, co-morbidities such as heart failure, chronic pain syndrome, metabolic disease, chronic obstructive pulmonary disease and/or neurological disorders (e.g. Parkinson’s). Participants were also excluded if they had undergone hip or knee replacement in the previous 2 years, or had been immobilised for greater than 1 week 3 months prior to testing. All the participants were community dwelling and socially active. Participants were not known to suffer from musculoskeletal or cardiovascular disease, any limb fractures within 5 years of testing and were classed as healthy.

**Table 1:** Participant characteristics

	Women (*n* = 17)			Men (*n* = 17)			Effects		
	Baseline	Follow-up	% Change	Baseline	Follow-up	% Change	Time	Sex	Sex*Time
Age (years)	71.1 ± 2.8	75.9 ± 2.6		71.3 ± 4.1	75.9 ± 4.4		*P* = 0.000	*P* = 0.924	*P* = 0.562
Body mass (kg)	65.8 ± 10.5	64.2 ± 11.3	− 2.74	84.2 ± 15.5	84.4 ± 15.4	0.24	*P* = 0.174	*P* = 0.000	*P* = 0.093
Height (m)	1.61 ± 0.07	1.59 ± 0.06	− 1.24	1.75 ± 0.07	1.74 ± 0.07	− 0.57	*P* = 0.000	*P* = 0.000	*P* = 0.774
BMI (kg·m^−2^)	25.8 ± 5.3	25.5 ± 5.6	− 1.16	27.6 ± 4.5	28.0 ± 4.20	1.45	*P* = 0.905	*P* = 0.186	*P* = 0.045
FFM (kg)	39.0 ± 3.0	37.9 ± 3.2	− 2.82	56.2 ± 7.37	55.2 ± 6.91	− 1.78	*P* = 0.002	*P* = 0.000	*P* = 0.861
FM (kg)	24.5 ± 9.6	24.1 ± 10.5	− 1.63	24.7 ± 10.5	25.8 ± 10.5	4.45	*P* = 0.376	*P* = 0.798	*P* = 0.107
FM (%)	37.4 ± 9.34	37.4 ± 10.0	0.00	29.4 ± 8.8	30.8 ± 8.5	4.76	*P* = 0.163	*P* = 0.025	*P* = 0.163
ALMM (kg)	15.8 ± 1.7	15.1 ± 1.6	− 4.43	24.1 ± 3.4	22.9 ± 3.1	− 4.98	*P* = 0.000	*P* = 0.000	*P* = 0.134
ALM·h^−2^ (kg·m^−2^)	6.05 ± 0.42	5.88 ± 0.40	− 2.81	7.87 ± 0.84	7.58 ± 0.65	− 3.68	*P* = 0.000	*P* = 0.000	*P* = 0.289
CSA_Quad_ (cm^2^)	44.2 ± 6.3	42.8 ± 5.9	− 3.17	64.6 ± 9.99	58.7 ± 8.0	− 9.13	*P* = 0.000	*P* = 0.000	*P* = 0.029
CSA_thigh_ (cm^2^)	94.0 ± 18.8	89.1 ± 17.2	− 5.21	125.7 ± 33.3	118.0 ± 32.6	− 5.60	*P* = 0.000	*P* = 0.005	*P* = 0.294

*BMI* body mass index, *FFM*: fat free mass, *FM*: fat mass, *ALMM*: appendicular lean muscle mass, *CSA*_*Quad*_: cross-sectional area quadriceps muscle; *CSA*_*thigh*_ cross-sectional area thigh

### Anthropometrics

The standing height of the participants was measured with a portable Stadiometer (SECA, Switzerland) to the nearest 0.1 cm. A digital scale (SECA, Switzerland) was used to record body mass to the nearest 100 g with participants wearing light indoor clothing. The body mass index (BMI) was calculated as body mass divided by height squared.

### DXA

Participants wore a medical gown and laid supine on the scanning bed. A total body DXA (Lunar Prodigy Advance, GE Healthcare, Chicago, USA) scan was performed to measure total body composition. Each total body scan took 295 s with an estimated skin entrance dose of 0.4 µGy (GE Healthcare, Lunar encore, Safety and Specification Manual). The system was calibrated with the same phantom at baseline and at 5 years follow-up before each scan. Off-line analysis (encore 2006 v 10.50.086) identified whole body lean mass and body fat percentage, arm and leg lean mass and bone mineral content (McPhee et al. 2013). Appendicular lean mass muscle mass (ALMM) was calculated as

(leg lean mass + arm lean mass) – (leg bone mineral content + arm bone mineral content) (Goodpaster et al. 2006).

### Magnetic resonance imaging (MRI)

Quadriceps and total thigh muscle volume was measured using a 0.25-T MRI scanner (G-Scan, Esaote, Genova, Italy). The participant was in a supine position in the scanner and multiple 3.1-mm-thick serial transverse sections were acquired every 25 mm from the proximal to the distal heads of the femur of the dominant leg, using a turbo 3D T1-weight protocol (matrix 256 × 256, TR 40 ms, TE 16 ms). The cross-sectional area of the quadriceps muscle (CSA_Quad_) and other thigh muscles (CSA_thigh_: hamstrings, abductors and adductors) in each slice were determined using computing imaging software (OsiriX medical imaging software, OsiriX, Atlanta, USA). The CSA_Quad_ was estimated using the maximal cross-sectional area from the serial transverse sections. CSA_thigh_ was estimated from the maximal cross-sectional area using previously outlined methods (Maden-Wilkinson et al. 2015; McPhee et al. 2009; Morse et al. 2007).

### Balance

Balance was determined as the time a person could stand on one leg, first with eyes open and then with eyes closed. The methods have been described previously (Maden-Wilkinson et al. 2015; McPhee et al. 2013). Participants were allowed three attempts at each condition (eyes open or eyes closed) and were allowed to select any leg to stand on. The arms were held close to the body, the standing leg was maintained in the same position and the resting leg lifted approximately 5 cm off the ground. If a participant achieved 30 s then there was no requirement to repeat the test.

### Six-minute walk distance (6MWD)

To assess the 6-min walk distance two cones were placed 20 m apart. Participants were given the verbal instruction to “complete as many circuits as possible without running” and received verbal encouragement after each minute of the walk. The total distance walked during the six-minute period was recorded (Enright 2003). Heart rate was monitored throughout the test (Polar, USA) and the average heart rate during the final 3 min of the test was given as the steady state heart rate (S-shr) (Maden-Wilkinson et al. 2015). All participants completed the 6-min walk without the use of a walking aid.

### Timed up-and-go (TUG)

The timed up-and-go (TUG) test involved getting up from a standardised chair (no arm rests, seat 44 cm high) and to walk forward as quickly as they were able, without running, to a cone 3 m away and return to the initial sitting position. Participants were familiarised to the procedure prior to the execution of the real test. Upon the ‘go’ signal, participants rose from the chair and timing was concluded when seated again. The test was conducted three times for each participant, with a rest period of 1 min between trials, and the quickest of the three trials was recorded.

### Muscle power

A maximal countermovement jump was performed on a force platform (Leonardo, Novotec Medical, Pforzheim, Germany) to measure the power of the leg extensor muscles. The participant was asked to perform the test three times, with a 1-min rest between jumps. The vertical component of the ground reaction force was used to calculate: jump height (m), maximal force (kN), maximum power of the concentric phase (Watts) and take-off velocity during the countermovement jump (V_CMJ_ in m·s^−1^) (Caserotti et al. 2001).

### Isometric maximal voluntary contraction (MVC) torque

Isometric knee extensions were performed with the right leg on a custom-made isometric testing dynamometer (Designed by the Department on Physical and Medical Technology, VU University, Amsterdam, The Netherlands). Force signals were recorded via customised Labview (National Instruments Corporation, Texas, USA) and Matlab software (Matlab, the Mathwork Inc, S Natik, MA, USA). All procedures were explained to the participants, emphasising the requirement to stay relaxed and only to voluntary contract when instructed to do so. The participants were seated on the dynamometer with a knee angle of 90° (full extension being 0°) and 85° hip flexion (supine being 0°). The lower leg of the participants was securely fastened to the force transducer, 2 cm above the ankle malleolus. The hip joint was firmly held in place via a strap. Prior to the measurements, the participants were familiarised to the knee extension exercise with three contractions at around 50% of maximal effort lasting 3 s each, followed by two further contractions at around 80% maximal effort lasting 3 s each. A two-minute rest was given prior to a maximal voluntary contraction (MVC) sustained for around 3 s. Two or more maximal contractions were performed until the two highest values were within 10%, with the highest value taken as MVC. Verbal encouragement and visual feedback were conveyed during the testing.

### Statistics

Data were analysed using SPSS v22 (IBM, 2015). The Shapiro–Wilk test was used to assess whether data were normally distributed. A mixed-model two-way ANOVA with Bonferroni post hoc test was used to examine differences over time and between sexes: Time (baseline *vs.* follow-up) was used as the “Within” Factor and Sex as the “Between” factor. To determine relationships between the dependent variables (6MWD and TUG) and independent variables, linear regression analysis was conducted. Data were expressed as mean ± standard deviation unless stated otherwise and differences were considered significant at *P* < 0.05.

## Results

### Body composition and physical function

Participant characteristics are shown in Table 1. Both sexes lost FFM over the 5 years of follow-up (*P* ≤ 0.001), but there was no significant change in FM or %FM. Appendicular lean muscle mass, sarcopenia index calculated as ALMM/h^2^, CSA_Quad_ and CSA_thigh_ were all lower at follow-up than at baseline (all *P* < 0.001), showing progression of muscle wasting. The average ALMM/h^2^ for older men (7.9 kg·m^−2^ baseline, 7.6 kg·m^−2^ follow-up) and women (6.1 kg·m^−2^ baseline and 5.9 kg·m^−2^ follow-up) were above the sarcopenia cut-off values of 7.0 kg·m^−2^ and 5.5 kg·m^−2^ for men and women, respectively (Cruz-Jentoft et al. 2019).

Balance decreased over the 5-year period as reflected by a shorter time the participants could stand on one leg with eyes open (Table 2; *P* < 0.001) or eyes closed (Table 2; *P* < 0.05). TUG and 6MWD performance decreased over the 5-year period (Table 2; all *P* < 0.05). The steady-state HR during the 6MWD was lower at follow-up than at baseline and this remained the case when expressed as %HR_max_ (Table 2; *P* ≤ 0.001).

**Table 2:** Measures of mobility

	Women (*n* = 17)			Men (*n* = 17)			Effects		
	Baseline	Follow-up	% Change	Baseline	Follow-up	% Change	Time	Sex	Sex*Time
1_leg_EO (s)	25.3 ± 8.0	17.8 ± 10.5	− 29.6	24.6 ± 9.2	15.2 ± 10.0	− 38.2	*P* = 0.000	*P* = 0.610	*P* = 0.554
1_leg_EC (s)	6.53 ± 5.34	3.31 ± 1.97	− 49.3	5.95 ± 5.21	3.93 ± 1.92	− 34.0	*P* = 0.015	*P* = 0.984	*P* = 0.562
TUG (s)	5.68 ± 0.59	7.07 ± 1.21	24.5	5.11 ± 0.59	6.01 ± 0.85	17.6	*P* = 0.000	*P* = 0.003	*P* = 0.150
6MWD (m)	533 ± 83	494 ± 69	− 7.3	568 ± 59	514 ± 59	− 9.5	*P* = 0.000	*P* = 0.226	P = 0.434
S-Shr	119 ± 12	113 ± 11	− 5.04	111 ± 17	104 ± 15	− 6.3	*P* = 0.000	*P* = 0.106	*P* = 0.759
6MWD % HR_max_	75.7 ± 10.1	73.2 ± 7.3	− 4.4	69.8 ± 11.1	66.6 ± 9.3	− 4.6	*P* = 0.001	*P* = 0.086	*P* = 0.665

*1*_*leg*_*EO* one leg balance eyes open, *1*_*leg*_*EC* one leg balance eyes closed, *TUG* timed up-and-go, *6MWD* six-minute walk distance, *S-Shr* six-minute walk steady state heart rate, 6MWD % *HR*_*max*_ six-minute walk percentage of maximum predicted heart rate

Isometric knee extensor MVC as well as countermovement jump power and V_CMJ_ all declined significantly over 5 years of follow-up (Table 3; *P* ≤ 0.005). There was no significant change in peak ground reaction force during the countermovement jump over 5 years of follow-up.

**Table 3:** Muscle function

	Women (*n* = 17)			Men (*n* = 17)			Effects		
	Baseline	Follow-up	% Change	Baseline	Follow-up	% Change	Time	Sex	Sex*Time
Standing jump power (W·kg^−1^)	23.7 ± 5.9	21.6 ± 5.0	− 8.86	29.9 ± 4.7	26.7 ± 4.8	− 10.70	*P* = 0.000	*P* = 0.003	*P* = 0.277
V_CMJ_ (m·s^−1^)	1.94 ± 0.19	1.81 ± 0.18	− 6.70	2.22 ± 0.19	2.18 ± 0.26	− 1.80	*P* = 0.009	*P* = 0.000	*P* = 0.171
Standing jump force (kN)	1.32 ± 0.32	1.38 ± 0.26	4.55	1.76 ± 0.40	1.82 ± 0.30	3.41	*P* = 0.166	*P* = 0.000	P = 0.978
Knee extensor MVC (N)	417 ± 72	366 ± 75	− 12.23	595 ± 83	518 ± 108	− 12.94	*P* = 0.000	*P* = 0.000	*P* = 0.344

*V*_*CMJ*_ take-off velocity during the countermovement jump, *MVC* maximal voluntary isometric contraction

### Correlations with 6MWD and TUG performance

Only at follow-up did balance, in terms of the time standing on one leg with eyes closed, correlate with the 6MWD (*R* = 0.47; *P* = 0.002) and TUG (*R* = 0.39; *P* = 0.01).

Both at baseline (*R* = 0.56; *P* < 0.001) and follow-up (*R* = 0.61; *P* < 0.001) the 6MWD correlated with power (Fig. 1A). The performance in the 6MWD did not correlate significantly with MVC·BM^−1^ (Fig. 1B), but did correlate with V_CMJ_ (Fig. 1C) at baseline (*R* = 0.57; *P* < 0.001) and follow-up (*R* = 0.57; *P* < 0.001).

Figure 1D shows a positive correlation (indicated by the negative slope) between power and performance of TUG at baseline (*R* = − 0.67; *P* < 0.001) and follow-up (*R* = − 0.53; *P* < 0.001). The MVC·BM^−1^ correlated to TUG at both baseline (*R* = − 0.30 *P* = 0.041) and follow-up (*R* = − 0.59 *P* = 0.000) (Fig. 1E). The performance of the TUG also correlated positively with V_CMJ_ (Fig. 1F) at baseline (*R* = − 0.71; *P* < 0.001) and follow-up (*R* = − 0.543; *P* < 0.005).

### Velocity and body mass

Figure 2 demonstrates that V_CMJ_ was inversely correlated with the BM·MVC^−1^ ratio both at baseline and follow-up (both *R* > 0.54; *P* < 0.001; Fig. 2). This relationship was not significantly changed over the 5-year period (Fig. 2). Given that the body mass did not change significantly over the 5-year period, this indicates that the main cause of a reduced V_CMJ_, and thus power, was that muscles were forced to work to the left side of the force–velocity relationship due to a reduction in MVC and not an intrinsic slowing of the muscle.

The higher V_CMJ_ in men than women (*P* < 0.001) was likewise attributable to a higher MVC rather than faster contractile properties in men than women. The % change in power was not significantly related to baseline power (Data not shown; *R* = 0.196 *P* = 0.133).

Note that one person performed poorly in both the 6MWD and TUG during follow-up (indicated with an arrow in all figures). This person had a steady-state heart rate in the normal range during the 6MWD, but low power. This low power was attributable to a reduction in the force generating capacity and not so much slowing of the muscle as the lower V_CMJ_ was as expected from the BM·MVC^−1^ for this person.

## Discussion

The novel observation in this longitudinal study was that healthy septuagenarians experience a significant decline in muscle mass/function and mobility over a 5-year period, irrespective of sex. The annual decline was larger than that observed in our previous cross-sectional study comparing 23- and 72-year-old people (Maden-Wilkinson et al. 2015), suggesting an accelerated age-related decline beyond the age of 70. Muscle power, determined with a countermovement jump, correlated most with performance in the 6-min walk and timed up-and-go tests, at both baseline and follow-up, while balance was associated with performance at follow-up only. The loss of power in the septuagenarians was primarily due to a reduction in force generating capacity, rather than a further slowing of the muscle. These results suggest that muscle power is a key determinant of physical function during relatively long- and short-duration physical function tasks and that with advancing older age, balance is of increasing importance for physical function.

### Decline in muscle function and physical function

The main defining features of sarcopenia are low muscle mass, weakness and reduced physical function (Cruz-Jentoft et al. 2010, 2019; Fried et al. 2001). The ALM/h^2^ were at baseline and follow-up above the sarcopenia cut-offs for men (7.0 kg·m^−2^) and women (5.5 kg·m^−2^) (Cruz-Jentoft et al. 2019), suggesting that our participants were not sarcopenic according to these cut-points. While an increase in TUG time is associated with an amplified risk of a falling, decline in physical function and an increase in frailty index (Beauchet et al. 2011; Kojima et al. 2015; Viccaro et al. 2011), the TUG time at follow-up was still (except the woman excluded from analysis) well below the 12-s cut-off point for normal mobility (Bischoff et al. 2003), suggesting they were not physically frail. Similarly, the 6MWD is commonly used to assess functional capacity (Enright and Sherrill 1998; Troosters et al. 1999). As with the TUG test, even though performance in the 6MWD decreased over the 5-year period, all participants (again except the excluded woman) covered ≥ 400 m at follow-up, considered the cut-off for mobility limitations (Abellan van Kan et al. 2011). Therefore, the participants in our study were not sarcopenic according to the cut-offs for skeletal muscle mass, nor (except one older woman at follow-up) physically frail, but rather a population of healthy ageing people.

### Accelerated decline

The annual reduction in 6MWD and TUG performance was larger in the present longitudinal study (0.9 and 4.2%, respectively) than that calculated from our cross-sectional study (0.4 and 0.7%) (Maden-Wilkinson et al. 2015). This and the annual decline in jumping power of 0.8% in our previous study (Maden-Wilkinson et al. 2015) compared to 2.0% in the present longitudinal study suggest an accelerated decline in functional capacity beyond the age of 70 years. These calculations assume that human peak performance occurs early in the third decade, something that has been observed in master athletes of several disciplines (Berthelot et al. 2012; Ganse et al. 2018). Therefore, our findings suggest an accelerated decline in muscle power in the 8th decade of life cannot solely be due to decreased physical activity levels, since similar declines are evident even in athletic populations (Degens 2012; Frontera et al. 2000, 2008; Lazarus and Harridge 2017; Ganse et al. 2018). If that decline continues to progress at the same, or an accelerated, rate it will ultimately result in a transition from an independent to a dependent lifestyle, even in our free-from-physical-limitations non-sarcopenic population. It is therefore important to uncover the ageing-related changes that elicit these deficits in functional capacity.

### Contribution of aerobic component and balance to the decline in 6MWD and TUG

The decreased performance in the TUG and 6MWD during follow-up correlated with a decline in balance, something also reported previously in people > 70 years (Chen and Chou 2017). Although even before the age of 70 a significant reduction in balance occurs (Onambele et al. 2006), the absence of significant correlations between balance, assessed as the duration one could stand on one leg with eyes closed, with 6MWD and TUG performance at baseline suggests that only after the balance impairment exceeds a certain threshold it becomes a limiting factor for daily life performance.

The 6MWD requires an aerobic component and is related to the maximal oxygen consumption of healthy young-adults and older people (Manttari et al. 2018). The heart rate measured during the 6MWD was decreased over the 5-year period both as absolute values and when expressed relative to the estimated age-predicted maximum. This reflects both a decrease in cardiovascular function and a decrease in the relative effort of the older adults when walking. It is not clear why less effort would be applied during the walk and our study methodology cannot reveal the reasons in any detail. However, it may be related to concerns over balance and the risk of falling. This supports previous studies of people aged > 70 years (Chen and Chou 2017) and suggests that deterioration of balance makes an increasing contribution to physical functional declines with advancing age, either directly as adjustments are made with each step to control posture, or indirectly through more caution due to fear of falling.

### Contribution of muscle function to the decline in 6MWD and TUG

Previous studies have shown that the performance in the 6MWD and TUG tests are related to muscle mass and function (Bijlsma et al. 2014; Janssen et al. 2002; Maden-Wilkinson et al. 2015; Song and Geyer 2018). However, the relationship between functional limitation and muscle mass in older people is weak (Lauretani et al. 2003) or even absent (Maden-Wilkinson et al. 2015) and we showed that power is more important. In a previous study we found that the ageing-related loss of muscle strength during early ageing is due to both a loss of muscle mass and quality, in terms of force generating capacity per unit muscle cross-sectional area, while in the 8^th^ decade of life it is primarily due to a loss of muscle mass (McPhee et al. 2018). It is thus possible that in the oldest-old a low muscle mass becomes an increasingly important contributor to reduced functional capacity. Yet, we found neither a significant relationship between muscle mass and functional capacity at baseline nor at 5-year follow-up. This confirms the increasing notion that not so much muscle mass, but rather muscle functional capacity is relevant as a determinant of the ability to perform daily life activities in the older population, even in people in the 8^th^ decade of life. Given that power is the product of force and velocity one can understand that muscle power has been reported to better correlate with functional capacity than maximal voluntary isometric force in the older population (Maden-Wilkinson et al. 2015; Reid and Fielding 2012). This argument was further supported in our longitudinal cohort at both baseline and 5-year follow-up.

We have previously shown that the difference in jump power between young and older subjects is in part due a reduction in force generating capacity and in part due to slower contractile properties of the muscle. While power increased with force, for a given jump force, power was greater in the young subjects (Maden-Wilkinson et al. 2015). This was attributed to a reduction in the intrinsic speed of shortening of the older muscle. Probably a consequence of an ageing-related fast-to-slow transition in fibre type composition (Larsson and Ansved 1995; Moreillon et al. 2019), preferential atrophy of fast fibres (Barnouin et al. 2017) and/or a slowing of type I and type IIa muscle fibres (Degens et al. 1998; Larsson et al. 1997),

However, when we looked further into this apparent slowing over the 5-year period, it appeared that there was no significant change in the body mass:maximal force ratio (Fig. 2). This means that both at baseline and follow-up, at a given ‘body mass:maximal force ratio’ the shortening velocity during the countermovement jump is the same. Given that body mass was not significantly changed over the 5-year period, but force was reduced by about 12%, the slower take-off velocity in the countermovement jump must have been the consequence of loss of force generating capacity. Thus, the actual culprit behind this apparent slowing of the muscle is that they are working at a higher relative load than 5 years prior, and therefore contracting slower according to the force–velocity relationship. Thus, while intrinsic slowing of the contractile properties was suggested to be the main determinant of the ageing-related reduction in jump velocity between 23 and 72 years of age (Maden-Wilkinson et al. 2015), it is the loss of force that causes a further decline in power in the ageing septuagenarian.

The interesting pattern therefore arises that during early ageing (23–72 years) particularly intrinsic slowing contributes to the loss of power (Maden-Wilkinson et al. 2015) and that weakening is the result of loss of muscle mass and quality (McPhee et al. 2018). On the other hand, during later ageing (71–76 years) loss of force, due to a loss of muscle mass and reduced voluntary activation (Degens et al. 2021; McPhee et al. 2018), but not further loss of muscle quality, is the primary contributor to the ageing-related loss of power.

As previously mentioned, this loss of power may eventually lead to a transition from an independent to a dependent life style. The cascade of these changes are in part to a number of factors such as the remodelling of the neuromuscular system which occurs in ageing through the loss of motor neurons (Piasecki et al. 2016), alongside the slowing of contractile properties (Yu et al. 2007). It has been shown, however, that muscle strength is positively related to physical activity levels in the older person (Latorre-Roman et al. 2016). An increase in physical activity levels may thus reverse the loss of strength in the older person and improve functional performance. Indeed, resistance exercise is a potent means to improve functional performance, even in the oldest old (Fiatarone et al. 1990).

Our study has a number limitations within the work. The participants recruited for the MYOAGE study (www.myoage.eu) (McPhee et al. 2013) did have physical activity measured at baseline and were classed as healthy and socially active but this was not measured at follow-up. Our cohort is representative of older community-dwelling adults and the sample size may appear small, though it is relatively large for an invasive longitudinal study.

## Conclusion

In conclusion, the ageing-related reduction in functional capacity over a 5-year period in healthy septuagenarians was to some extent attributable to a reduction in maximal heart rate and balance within this particular cohort. However, a larger proportion of the decline in performance during 6-min walk and timed up-and-go tests was explicable by a decline in muscle power. In contrast to the intrinsic slowing of the muscle between 23 and 72 years of age (Maden-Wilkinson et al. 2015), the further decrement in power and performance of the functional tests between 71 and 76 years of age was primarily attributable to loss of strength. This suggest that the process of muscle ageing may change from decrements in both muscle mass and quality during early ageing to principally a reduction of muscle mass during later stages of ageing.

## Data Availability

Data available on request from the authors.

## Abbreviations

6MWD: 6-Minute walk distance
MVC: Maximal knee extensor strength
TUG: Timed up-and-go
MRI: Magnetic resonance imaging
DXA: Body dual-energy X-ray absorptiometry
ALMM: Appendicular lean mass muscle mass
CSA_Quad_: Cross-sectional area of the quadriceps muscle
CSA_thigh_: Other thigh muscles
V_CMJ_: Take-off velocity during the countermovement jump
FFM: Fat free mass
FM: Fat mass
HR: Heart rate
